# An Analysis of Media Usage in a Surgical Education Program

**DOI:** 10.7759/cureus.83801

**Published:** 2025-05-09

**Authors:** Obinna Enemoh, Bernard O Chukwumah, Stephen O Agboro

**Affiliations:** 1 General Surgery, Chesterfield Royal Hospital, Chesterfield, GBR; 2 General Practice, New Cross Hospital, Wolverhampton, GBR; 3 General Surgery, University Hospital Lewisham, London, GBR

**Keywords:** educator training program, media education, medical education, online teaching, post graduate medical education, social media, specialty training

## Abstract

Introduction

In the United Kingdom (UK), the General Medical Council (GMC), the regulatory authority for doctors, emphasizes teaching competence in its Good Medical Practice guidelines. Teaching experience is also a key criterion in core and specialty training applications. In response to these expectations, we established the Surgical trainee interest group (STIG), a collaborative platform for junior doctors focused on addressing training needs, particularly in teaching. Through STIG, we developed and implemented a peer-led education program aimed at enhancing teaching skills and supporting the development of individual teaching portfolios. The program included quarterly teaching sessions on surgical topics and preparatory courses for specialty applications, conducted over one year (2022-2023).

Traditional teaching programs are often resource-intensive and challenging to organize. Media can help bridge this gap by enhancing teaching delivery, improving learner comprehension and retention, and fostering collaboration through broader access to educational opportunities. In our program, teaching sessions were delivered online using platforms such as Medall and Microsoft Teams. Recruitment and dissemination of activities were primarily conducted via social media.

This study aimed to evaluate the effectiveness and challenges of integrating media into a surgical teaching program for resident doctors. Given the limited literature on media use in surgical education, our research provides valuable insights into the advantages and limitations of leveraging digital platforms for medical teaching.

Methods

Resident doctors who participated in the teaching program were asked to complete an online feedback form at the end of each session. The form included questions assessing the effectiveness of media integration within the teaching program. Only participants who provided consent and submitted the feedback form were included in the analysis; no exclusion criteria were applied. Quantitative feedback was collected using a 5-point Likert scale, and a comments section was provided for qualitative feedback. To explore perspectives on media usage, semi-structured interviews were conducted with the tutors. All data were subsequently collected and analyzed.

Results

Forty participants attended our teaching sessions on the Medall platform, while 12 attended via Microsoft Teams. The on-demand content from our sessions recorded a total watch time of 784 minutes. Respondents rated the use of media with an average score of 4.5 on a 5-point Likert scale. Many respondents agreed that the use of media was effective in supporting teaching delivery. Positively themed comments were noted, while challenges and areas for improvement were also identified. Tutors reported that media enhanced their ability to deliver effective teaching and supported the overall objectives of the program.

Conclusion

Our analysis highlights the role of media in medical education. We evaluated the effectiveness and challenges associated with media integration in a surgical teaching program. The study demonstrated that media, when integrated into surgical education, is effective in enhancing teaching delivery. While the pivotal role of media in instruction was evident, several challenges were identified that warrant further improvement.

## Introduction

Doctors undergo post-graduate training to gain skills and competencies in different specialties. The General Medical Council (GMC) regulates education for medical students and post-graduate training for doctors in the United Kingdom (UK). It expects medical professionals to be competent in teaching, as outlined in the council's Good Medical Practice guidelines [1]. Teaching experience is a key criterion in both core and specialty training applications, prompting practitioners to engage in educational activities as learners and educators. Entry into all training programs remains highly competitive, with competition ratios for training posts steadily increasing over the past five years [2]. International medical graduates (IMGs), in particular, face significant challenges in progressing to surgical training, often requiring considerable time and support to develop a competitive portfolio [3]. To address this, we established the Surgical Trainee Interest Group (STIG) as a collaborative platform for junior doctors, with a focus on supporting their training needs, particularly in teaching. This peer-led education initiative specifically targeted IMGs interested in surgery, aiming to enhance their teaching competence and facilitate the development of a teaching portfolio for national specialty applications, framed as a quality improvement project.

Medical education comprises basic medical education, specialist training, and continuing medical education. It is an evolving specialty that uses scientific, clinical, and social principles to meet the training needs of a dynamic healthcare profession and the society at large [4]. Traditionally, medical education was done only through in-person teaching, books, and journals. However, with advances in technology, the internet has facilitated connectivity and information transfer to a wider audience. Media, in the context of this article, refers to teaching aids which facilitate the transfer of knowledge and information to the end users [5]. This new media has a significant impact on learning because it is flexible to use, and fosters collaboration [6]. Indeed, evidence suggests these digital technologies have changed the landscape of education and led to improved access to teaching [7]. For our education program, we hosted teaching sessions on online platforms like Medall and Microsoft Teams, while recruitment to our group and publicity for our activities was via social media. This use of media provided trainees access to teaching and skills development.

We analyzed the use of media in our education program. The aim of this study was to evaluate the effectiveness and challenges of media integration in a surgical teaching program for resident doctors. Other outcomes were to highlight the role of media in the organization and delivery of an education program; demonstrate media as a tool to develop teaching skills, and check learner satisfaction in the delivery. There is paucity of literature on the evaluation of media usage in a surgical education program, and this study will throw the much-needed light on the merits and challenges of the use of social media in medical teaching. 

## Materials and methods

Surgical trainee interest group (STIG) 

The surgical trainee interest group (STIG) is made up of resident doctors with an interest in surgery and working in the UK. It provides a platform for peer teaching and educational development. Our supervisors provided teaching support to the tutors either physically, via emails, or through other online resources. Teaching sessions were observed by either a consultant or a trainee from a higher specialty. Our program had quarterly teaching sessions which ran for a year between 2022 and 2023. These consisted of five surgical teaching sessions on select surgical topics in the lecture format. Appendix 1 lists the topics covered in our program. We also hosted a core surgical training (CST) and a higher specialty training preparatory course using the webinar format. Teaching sessions were hosted via online platforms such as Medall (MedAll Inc., Belfast, Northern Ireland) and Microsoft Teams (Microsoft Corp., Redmond, WA, US). Medall recorded our sessions and uploaded them online as catch-up content, while Teams did not have a similar feature. 

Study design

A web-based feedback survey was administered to all participants at the end of each teaching session. This was administered prospectively throughout the program, and it collected quantitative and qualitative data on media usage. From their website, Medall sent the feedback forms to all participants after each session via email, while we shared feedback forms generated by Microsoft (Microsoft Corp., Redmond, WA, US) after our Microsoft Teams sessions. This feedback was collected anonymously and analyzed. We did not find any previously validated questionnaires, so we created one for our survey. The questions asked were: (1) How effective do you find the use of media in organizing this teaching program? (2) How do you find this platform used for delivering this session? The answers were recorded quantitatively on a 5-point Likert scale. A score of one indicated poor use of media while a score of five showed excellent use of media for learning. We found an average of the scales to give us a quantitative score. Qualitatively, our feedback included a suggestion box on the use of media, and included the questions: (1) What went well? and (2) What could have been better? Semi-structured interviews were conducted by the program facilitators with the tutors to assess their perspectives on the use of media in enhancing teaching opportunities for trainees. This analysis did not require ethical approval because the education program is registered as a quality improvement project. No medical or identifiable data was collected. 

Study participants 

All participants were resident doctors working in the UK. Four tutors shared their opinions on the subject via semi-structured interviews. Participants who consented and submitted a feedback form were included; there were no exclusion criteria. 

Data analysis 

Medall provided analytical data including quantitative scores for feedback, comments, performance data on catch-up content, and views. Our feedback form on Teams was analyzed using Microsoft Excel (Microsoft Corp., Redmond, WA, US). The comments on the feedback forms and the opinions from the structured interviews were analyzed using a thematic analysis methodology [8]. This was done by two of the authors, who acted independently initially, and identified themes. Emergent codes became apparent on analysis, and these were refined and mapped into positive or critical themes. 

## Results

Forty-five participants attended our teaching sessions on Medall, while 12 attended our session on Teams. The on-demand content from our teaching session recorded a total watch time of 784 minutes. All participants (100%) joining synchronously completed a feedback form, while no one viewing the catch-up content provided feedback. An analysis of the effectiveness of our use of media, 45 respondents revealed an average score of 4.5. This demonstrated that most of the participants found the use of media as an effective tool to organize an education program, and it enhanced teaching delivery. 

The feedback and the semi-structured interview comments were analyzed using a thematic analysis methodology. We identified words and phrases which were positively themed and others that were more critical. We identified positively-themed words in the responses to our question in the suggestion box on what went well. Some of the positive comments on the effectiveness of media usage are listed in Table 1. 

**Table 1:** Comments by respondents including positively-themed words

Comments by respondents
Engaging
Awesome
Appropriate
Amazing
Interactive

Responses to the question on what could have been better were critical of the use of media, and are given in Table 2.

**Table 2:** Responses to the question on what could have been better

Comments by respondents
The technical aspects of the presentations
The Medall platform did not allow for an interactive session
Presentation-wise, ability to point out items on the screen with the cursor
The audio was a bit shaky
I was looking forward to seeing the urology presentation but because of technical problems it was not able to go ahead

The opinions of the tutors recorded from our semi-structured interviews are given in Table 3. 

**Table 3:** Opinions of the tutors on the use of media in our education program

Views of tutors on the use of media (recorded during interviews)
“This teaching would have been difficult to organize if not for media. Social media played a big role in sharing the message but I think that Medall platform was brilliant. It just seems to sort everything.”
“The most important use for me is the flexibility and easy access. As a trainee, this saves on travel and accommodation cost. I can arrange a teaching program for a decent number of people without so much stress.”
“Once the learners are happy, I am happy. It makes everyone happy.”
“We would have struggled to organize this teaching program using the traditional teaching approach. Getting a teaching space and learners in one place would have been a logistical nightmare. Media has its own challenges, but it seems to make these things smoother.”

## Discussion

Peer-led teaching is valuable to learners due to the understanding between the learners and the faculty [9]. Different strategies are being used to make this teaching method accessible to trainees for teaching development. We organized an education program for doctors interested in surgery to improve their teaching competence and build a teaching portfolio. In traditional teaching using a didactic approach, materials like a venue, audio-visual equipment, and posters are required to organize a teaching program. Tutors and learners are required to attend the session in person, which entails implications related to cost, time, and logistics. This makes organizing a teaching program challenging. The COVID-19 pandemic caused a significant disruption in medical education. Since then, an important educational innovation for teaching delivery is the use of media [10]. Media is known to bridge this gap due to its flexibility and easy access. The use of media in education has made it easier to organize teaching remotely with an effect similar to a traditional approach. This includes online live teaching infrastructure, asynchronous media, text, and visual aids. In this study, we shared the experience from our education program. The use of media provided trainees with access to a teaching platform through which they developed their skills and gained competence. This analysis of media usage underscores its role in enhancing information delivery while also acknowledging certain challenges.

We adopted synchronous teaching strategies using live video conferencing hosted on Medall and Microsoft Teams. Our teaching facilitators interacted with learners using PowerPoint (Microsoft Corp., Redmond, WA, US) presentations, video, audio, and a chat feature. Learners asked questions in real time, and tutors could check for understanding and adjust their teaching technique [11]. During our teaching sessions, the chat box feature was actively used by learners to ask questions that were responded to in real time. Video recordings of our live sessions were uploaded as on-demand videos on the MedAll website. The use of media in this manner has transformed the education landscape because asynchronous video tools allow learners to learn at their own pace and convenience [12]. This is especially useful for self-paced learning and continuous professional development. The use of social media platforms also increased interactions between surgical trainees across different regions in the country and other parts of the world. This created a medium for the exchange of ideas and for networking. Trainees could access research work from different sources, share ideas, and collaborate.

Medall

MedAll is a rapidly growing online platform utilized by medical professionals to host live events, video-based micro-learning sessions, conferences, and on-demand courses. Our teaching series was conducted using the MedAll platform, which provided tutors with a unique opportunity to integrate various forms of media into their teaching. It enabled remote participation for learners, generated reports on content performance, facilitated feedback collection and data analytics, and issued attendance certificates. Previously, these functions were managed through multiple web-based applications; however, MedAll consolidated them into a single website. This proved cost-effective, as we utilized the free version, and it streamlined the logistical management of our educational program. Our overall experience with the platform was mixed. While it offers valuable features such as data tracking, feedback facilitation, and certificate generation, we encountered some technical issues. A few participants reported audio problems, and one session had to be rescheduled due to a tutor’s inability to access the website.

Visual aids 

All our presenters prepared their sessions using PowerPoint, incorporating a range of visual aids to convey their information to the audience. Visual aids serve to reinforce key points in a presentation, engage the audience, and enhance information retention [13]. Multimedia platforms were used to display text, charts, graphs, images, and videos, aiding learners in comprehending the presented material.

Social media 

Social media has proven to be a highly effective tool in medical meetings for disseminating information about our activities and recruiting new members. A WhatsApp (WhatsApp, California, US) group, initially created by our founders, was later opened to interested participants following each teaching session. This platform was used to circulate teaching schedules and other educational resources. It has since been successfully integrated into other remote learning initiatives [14]. Additional social media platforms such as Facebook (Meta, California, US), X (formerly Twitter, Texas, US), and TikTok (ByteDance, Beijing, China) were also employed for member recruitment and for publicizing our activities. These efforts facilitated greater collaboration and contributed to the expansion of our professional networks.

Given the demanding schedules of our members, media platforms supported teaching efforts, enabled efficient information sharing with learners, and fostered interaction in a convenient, cost-effective, and accessible manner. Feedback on the teaching content was largely positive, though some suggestions for improvement were noted. Nevertheless, the use of media in education presents certain challenges. Media use in higher education has been linked to increased distractions, with concentration often compromised due to the demands of multitasking and the integration of multimedia formats [15]. This can be overwhelming, particularly for learners who struggle to maintain focus. Technical issues such as livestream disruptions, faulty video or audio presentations, hardware malfunctions, or poor internet connectivity also posed challenges [16]. These issues occasionally led to the cancellation of teaching sessions. Additionally, all patient data used in presentations must be anonymized in compliance with regulatory standards. Video conferencing must avoid violations of copyright laws related to media use. Proper permissions and consent are also required for filming or photographing delegates and presenters [17].

To enhance learning outcomes and the use of media, it is essential to continuously collect data on participant engagement, learner metrics, and feedback for thorough evaluation. This data must be assessed against established standards to enable organizers to draw meaningful insights and enhance the learner experience in future programs. These challenges were observed in our teaching program and were addressed accordingly. Our members will be applying to various specialty positions with teaching portfolios developed through this program. The integration of media provided a distinctive opportunity for them to cultivate and refine their teaching skills.

We acknowledge several limitations in our study. The small sample size and the focus on a single educational program is a significant constraint. Consequently, the findings may not be generalizable to larger programs or other regions. Future studies could explore similar analyzes in larger programs with more participants. While participants who attended the sessions synchronously provided feedback, no responses were received from those who viewed the catch-up content. The reasons for this are unclear, and further research is needed to investigate this gap. Additionally, participants with a pre-existing interest in teaching may have introduced response bias. Similarly, the involvement of program facilitators in conducting the semi-structured interviews could have influenced participant responses, introducing a potential source of bias. 

## Conclusions

Teaching competence is a vital skill expected of medical professionals. This is assessed in UK training applications. We organized a peer-led surgical education program for members to collaborate, improve their teaching experience, and prepare a portfolio for training applications. Media is a useful tool to improve the effectiveness of teaching and increase access to education at a reduced cost. Our program was hosted online using digital infrastructure and publicized via social media. Overall, our analysis underscores the role of media in medical education. We evaluated the effectiveness and challenges associated with integrating media into a surgical teaching program. The study demonstrated that the use of media, when incorporated into surgical education, is effective in enhancing teaching delivery. While the key role of media in instruction is evident, the study also identified challenges that warrant further improvement.

## Appendices

**Table 4:** Appendix 1: Topics covered in the education program

Topics covered in the education program
Acute abdomen
Basic principles in managing traumatic chest injuries
Principles of skin grafting
Basic surgical emergencies
Organ transplantation
Inflammatory bowel disease
Core Surgical Training application course
Urology ST3 application course
